# Identifying Reducing and Capping Sites of Protein-Encapsulated Gold Nanoclusters

**DOI:** 10.3390/molecules24081630

**Published:** 2019-04-25

**Authors:** Yu-Chen Hsu, Mei-Jou Hung, Yi-An Chen, Tsu-Fan Wang, Ying-Ru Ou, Shu-Hui Chen

**Affiliations:** 1 Department of Chemistry, National Cheng Kung University, Tainan 70101, Taiwan; f83712@gmail.com (Y.-C.H.); mr.comicchild@gmail.com (M.-J.H.); jelly601095@gmail.com (Y.-A.C.); r910061@gmail.com (Y.-R.O.); 2 Department of Applied Chemistry, National Chia-Yi University, Chia-Yi 60004, Taiwan; tfwang@mail.ncyu.edu.tw

**Keywords:** gold nanocluster, bovine serum albumin, protein, mass spectrometry, encapsulation, nucleation sites, biomineralization

## Abstract

The reducing and capping sites along with their local structure impact photo properties of the red bovine serum albumin-capped Au nanocluster (BSA-AuNC), however, they are hard to identify. We developped a workflow and relevant techniques using mass spectrometry (MS) to identify the reducing and capping sites of BSA-AuNCs involved in their formation and fluorescence. Digestion without disulfide cleavages yielded an Au core fraction exhibiting red fluorescence and [Au*_n_*S*_m_*] ion signals and a non-core fraction exhibiting neither of them. The core fraction was identified to mainly be comprised of peptides containing cysteine residues. The fluorescence and [Au*_n_*S*_m_*] signals were quenched by tris(2-carboxyethyl)phosphine, confirming that disulfide groups were required for nanocluster stabilization and fluorescence. By MS sequencing, the disulfide pairs, C75–C91/C90–C101 in domain IA, C315–C360/C359–C368 in domain IIB, and C513–C558/C557–C566 in domain IIIB, were identified to be main capping sites of red AuNCs. Peptides containing oxidized cysteines (sulfinic or cysteic acid) were identified as reducing sites mainly in the non-core fraction, suggesting that disulfide cleavages by oxidization and conformational changes contributed to the subsequent growth of nanoclusters at nearby intact disulfide pairs. This is the first report on precise identification of the reducing and capping sites of BSA-AuNCs.

## 1. Introduction

Proteins have been implicated as the primary active biomolecules involved in biomineralization [1,2,3]. Protein scaffolds provide unique metal coordination environments that promote biomineralization processes [4,5]. Fluorescent gold nanoclusters (AuNCs) encapsulated by bovine serum albumin (BSA) [6] have attracted considerable attention in recent years for their unique properties as new fluorescent probes in biological sensing [7,8,9], imaging [10,11,12], and biomedicine [13,14,15,16,17,18]. BSA molecules not only reduced the chloroaurate ions, but also directed the oriented growth of gold nanocrystals. The different shapes of gold nanoclusters were synthesized based on the template of different pH-dependent isoforms, but only the aged isoform (pH 12) yields large nanoclusters with red fluorescence [19,20]. Unlike a smaller close shell structure of nanoclusters (such as 8AuNC) in which emission energy follows the Fermi scaling rule and is solely dependent on their size [21,22,23], encapsulating ligands of large AuNCs have impacts on their fluorescence properties [24]. Moreover, the internal, and potentially cascaded, energy transfers (FRET) between nanoclusters and aromatic AAs of proteins such as tryptophan further enhance fluorescence [25]. Hence, controlling the nanoclusters with atomic precision and solving their local structures are critically important for improving the synthesis and application of red BSA-AuNCs. Nevertheless, the formation, structure, and fluorescence mechanism of BSA-AuNCs remain largely unknown.

The formation of Au nanoclusters was believed to involve the initial complexation of protein−Au ions. A further pH increase to 12 triggered the reduction of the Au complexes to Au atoms, accompanied with conformational conversion from a Normal (N) to Aged (A) isoform to accommodate the growth of AuNCs [6,26]. The tyrosine residue was described to be responsible for the reduction of the gold salt to the Au atom [6], since the reaction requires a high pH which correlates with the side chain pKa (10.46) of tyrosine. However, the transition from neutral to basic was investigated by Dixon et al. [20] and the threshold of the occurrence of red fluorescence was estimated to be at pH 9.7 ± 0.2, indicating that the hydroxyl (OH) group of tyrosine is not at a high enough pH to reduce the Au ion to Au(0). On the other hand, cysteine disulfide bonds were proved to be required for red fluorescence [20]. It has been suggested, but not verified, that the nucleation sites of BSA-AuNCs were also cysteine disulfide bonds [6]. Although single AuNC in BSA was reported based on the detection of gold nanocluster composites using advanced electron microscope; [27] multiple nucleation sites were still proposed based on the excitation-emission map [20] as well as molecular simulation [28]. Moreover, it is controversal whether the red fluorescence is due to the Au(0)NC core or metal ions complexed with BSA [20]. Understanding the local Au interaction with the AA residues and the sequence motifs will help shed light on these issues but they are hard to acquire by current techniques commonly used in nano science.

Protein mass spectrometry (MS) using matrix-assisted laser desorption ionization (MALDI) or electrospray ionization (ESI) coupled with liquid chromatography (LC) and proteomics are tools to resolve the sequence and modification sites in a protein. So far, MALDI-MS has become the main method to determine the number of encapsulated Au in a BSA by intact molecular weight measurement [20,29,30,31]. However, stringent requirements for sample preparation have challenged applications using MS-based detection. The extraction and interpretation of the resulting data are other challenges in proteomics. In this study, we intended to elucidate the core ligand sequence and key residues responsible for AuNC formation and stabilization using MALDI-MS, LC-MS^2^, and proteomics by developing a proper workflow and sample preparation method.

## 2. Results and Discussion

### 2.1. Characterization of As-synthesized BSA-AuNCs

**Figure 1:**
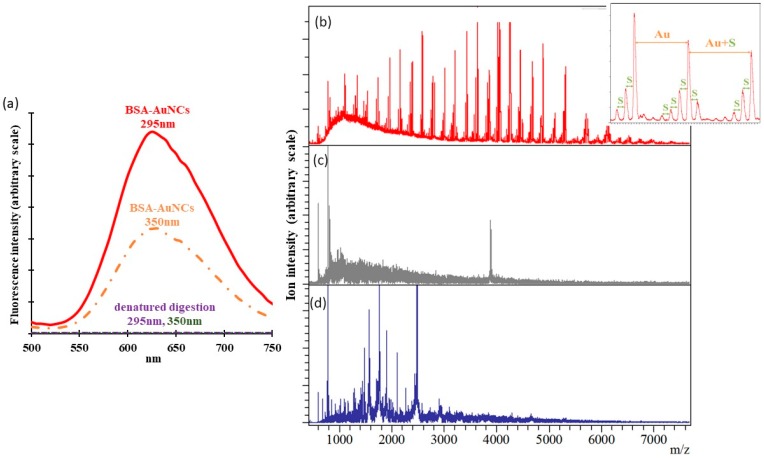
(**a**) Fluorescence of BSA-AuNCs and denature-digested BSA-AuNCs excited at 295 or 350 nm. [Au*_n_*S*_m_*] signals in the low mass region of MALDI-MS detected from (**b**) BSA-AuNCs solution, (**c**) BSA solution, and (**d**) denature-digested BSA-AuNCs. The enlarged spectra pattern of (**b**) is shown in the inset.

The synthesized BSA-AuNCs exhibited red (650 nm) fluorescence under λ = 350 nm excitation (Figure 1a). According to previous reports [20,29,30,31,32], the red fluorescence was attributed to the large BSA-AuNC containing 25 Au atoms (BSA-25 Au). Moreover, the red fluorescence under λ = 295 nm excitation was about two times stronger than that under 350 nm excitation (Figure 1a), suggesting fluorescence resonance energy transfer (*FRET*) was the dominant route of fluorescence [25,33]. The occurrence of FRET with BSA-Au-NC was attributed to the interaction between tryptophan (excited at 295 nm and emitted at 340 nm) and AuNC as addressed in the literature [25,33] based on a significant decrease in intensity and fluorescence lifetime of tryptophan when both were excited at 295 nm [33]. X-ray photoelectron spectroscopy (XPS) (Figure S1) indicated two distinct doublets, one with the Au 4f7/2 peak at 84.2 eV and the other at 85.2 eV, assigned to Au (0) (65–70%) and Au (I) (30–35%). These results were consistent with many reports [6,23,34].

**Scheme 1:**
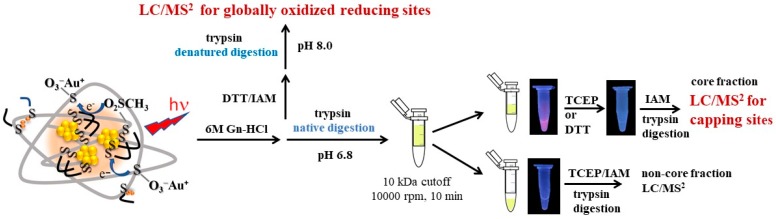
Sample preparation for bottom-up proteomics analysis of BSA-AuNC as well as its core and non-core fractions by LC/MS^2^.

MALDI-MS spectra of the red solution (Supporting information Figure S2) showed a broad peak related to BSA-25 Au at a maximum of ~71.3 kDa, as previously reported [6,35]. However, the exact number of Au was not clear since the peak width was as large as 1 kDa. It is also unknown whether there were single or multiple gold clusters in one BSA. In the low mass region (Figure 1b), we observed a distribution of negative ions having periodic spacing starting at *m*/*z* = 6000 (roughly corresponding to 25 Au), until it was impossible to resolve the spacing. These signals were not observed for the BSA (Figure 1c) solution and could be due to the fast heating of the gold core, followed by selective cleavage of the S-C bond under UV irradiation to eject the bulk gold [36]. Such signals have also been observed in transferrin-encapsulated AuNCs [37]. The ions detected can be described as having major and minor *m*/*z* spacing (inset in Figure 1b), consistent with the composition of the metal cluster core [38]. The minor periodic peaks in each group of ions correspond to an *m*/*z* = 32 spacing of sulfur atoms (*m* × S). The major *m*/*z* spacing between the most abundant ion in each adjacent group corresponds to a difference of either 197 (Au) or 229 (AuS), which can be described with the general formula [Au*_n_*S*_m_*]. These data indicated the AuNCs were encapsulated by functional groups containing sulfur atoms such as methionine, cysteine thiol, or disulfide ligands. The [Au*_n_*S*_m_*] signals were still detectable when the solution was filtrated using a 30-kDa membrane (data not shown), indicating that they were nanocluster ions ablated from large encapsulating molecules (> 30 kDa), rather than small gold ions or clusters in the solution. A workflow (Scheme 1) was then designed to identify reducing and capping sites on BSA for AuNCs formation.

### 2.2. Identification of Global Reducing Sites

For identifying reducing sites (up arrow of Scheme 1), two controls composed of BSA solutions at pH 7 and pH 12, were processed simultaneously with the red BSA-AuNC solution. Bottom-up proteomics was used to identify potential AAs oxidized upon Au ion reduction [39], including cysteine, histidine, methionine, and tryptophan, which have lower pKa values, as well as tyrosine. Two controls composed of BSA solutions at pH 7 and pH 12, were processed simultaneously with the red BSA-AuNC solution. Each solution was denatured by DTT, alkylated by IAM, and then digested with trypsin (Scheme 1). Upon denaturation and digestion (Scheme 1), the red fluorescence (Figure 1a), intact mass at 71.3 kDa (Supporting information Figure S2) and [Au*_n_*S*_m_*] signals (Figure 1d) disappeared, indicating that the denatured digestion was able to disintegrate the BSA-AuNCs and release the capping ligands. Only tryptic peptides of BSA were detected at a mass range < 2500 Da (Figure 1d and Figure S3e). This was thus deemed as a suitable sample preparation for proteomics analysis.

**Figure 2:**
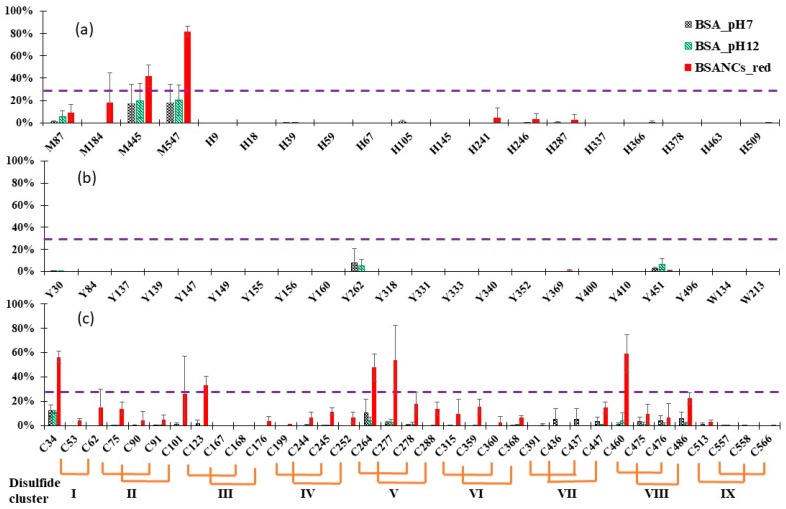
Global oxidization percentage of all (**a**) methionine and histidine residues, (**b**) tyrosine, tryptophan, and (**c**) cysteine residues of BSA, detected from BSA solution under pH 7 and pH 12, as well as BSA-AuNC solution. Each data point was the average of three independent measurements with ±1 standard deviation (error bar). Connections of disulfide pairs are indicated in the bottom of (**c**).

A near whole (97%) sequence of BSA was mapped by accurate mass (10 ppm) and MS^2^ fragmentation with an ion score > 20 (Supporting information Table S1) from the red BSA-AuNC solution. As shown (Table S1), multiple oxidization sites on methionine, histidine, tyrosine, and cysteine were identified. Assuming the ionization efficiency was similar for a modified and non-modified peptide pair, the global oxidization percentage of a specific site was calculated by dividing the peak intensity of all oxidized peptide sequences covering the same oxidization site, by the sum of peak intensity of the corresponding un-oxidized and oxidized peptides [40]. As shown (Figure 2), both the controls had either no detectable oxidization or oxidization percentage <20% for methionine sites, <5% for histidine, <10% for tyrosine, and <15% for cysteine sites. Increasing the pH from 7 to 12 did not significantly increase the oxidization percentage. Compared to the two controls, the red BSA-AuNC solution was extensively oxidized at multiple methionine and cysteine sites and a small percentage at some histidine sites (Figure 2), indicating their oxidization was induced by the Au ions. Among them, the oxidization at C34 in domain IA; C123 in domain IB; C264 and C277 in domain IIA; and M445, M547, and C460 in domain IIIA of BSA (annotated MS2 shown in Figures S4A–S4G) were determined to be largely oxidized (oxidization percentage > 30%). In contrast, the tyrosine residues were identified to have either no detectable oxidization or low (<5%) oxidization levels (Y262 and Y451), in not only the two controls, but also the BSA-AuNC solution, indicating that tyrosine may not be responsible for the reduction of Au ion as previously proposed [6]. We believe the requirement of basic condition (pH > 10) for red fluorescence was mainly for the conformational changes from N to A isoform, since only the A-isoform yielded red fluorescence. Instead, based on our data, cysteine and methionine were found to be the main residues responsible to trigger the reduction of Au ion complexes to Au atoms.

### 2.3. Disulfide Cleavages by Extensive Cysteine Oxidization

It is notable that almost all cysteines were extensively oxidized to sulfinic (SO_2_H) or sulfonic (SO_3_H) acid (Table S1), which must accompany disulfide cleavages. In addition to the only free thiol, C34, in domain IA, there are nine disulfide clusters (Figure 2c) in BSA [41,42]: one single disulfide bond (Cluster I: C53−C62 in domain IA) and eight disulfide pairs (Cluster II: C75−C91/C90−C101 in domain IA; Cluster III: C123−C168/C167−C176 in domain IB; Cluster IV: C199−C245/ C244−C252 and Cluster V: C264−C278/C277−C288 in domain IIA; Cluster VI: C315−C360/C359−C368 in domain IIB; Cluster VII: C391−C437/C436−C447; and Cluster VIII: C460−C476/ C475−C486 in domain IIIA; Cluster IX: C513−C558/C557−C566 in domain IIIB). Except for the free C34 in domain IA, the largely oxidized cysteines C123 and C460 belong to disulfide clusters III in domain IB and VIII in domain IIIA, respectively, while C264 and C277 belong to disulfide cluster V in domain IIA. Thus, as pH increased to 12, disulfide cleavages occurred simultaneously upon extensive oxidization on these cysteine sites, which coupled with the conformational changes from N- to A-isoform. At pH 12, it is generally believed that disulfide bonds become accessible, allowing for the formation of stable S−Au−S motif-capped Au nanoclusters in the protein template [28]. So far, however, experimental data are lacking to reveal which sites or residues are the capping ligands of AuNCs.

### 2.4. Identification of the Au-Core Capping Sites

**Figure 3:**
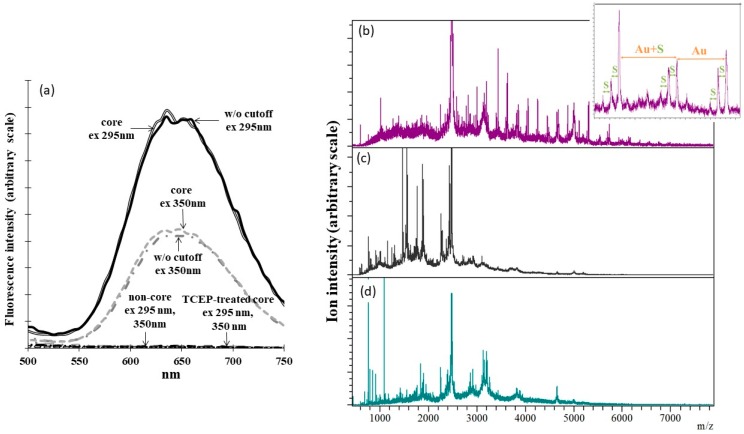
(**a**) Fluorescence of the native-digested BSA-AuNCs (w/o cutoff) and its core and non-core fraction separated using 10 kDa-cutoff membrane, as well as its disulfide-cleaved (by TCEP) core fraction excited at 295 nm or 350 nm. [Au*_n_*S*_m_*] signals in the low-mass region of MALDI-MS detected from the (**b**) core and (**c**) non-core fraction of native-digested BSA-AuNCs, as well as (**d**) disulfide-cleaved (by TCEP) core fraction. The spectra pattern of (**b**) was enlarged in the inset.

For identifying capping sites (right arrow of Scheme 1), we further performed trypsin digestion without DTT (native digestion) to conserve the disulfide linkages in BSA-AuNCs. In contrast to denatured digestion (Figure 1d), the native digestion yielded solutions that still exhibited red fluorescence at 650 nm excited by 295 nm (Figure 3a or Figure S3a) or 350 nm (Figure 3a or Figure S3b). Compared to intact BSA-AuNCs, the fluorescence intensity was reduced by 25% (Figure S3a,b) upon native digestion. Moreover, the fluorescence was quenched (Figure S3a,b) upon disulfide cleavage by TCEP, which is consistent with the report that the disulfide group is required for red fluorescence [20]. In the high-mass region, the intact mass signal (71.3 kDa) of BSA-AuNC disappeared (data not shown) upon native digestion. In the low-mass region, however, bulk gold signals [Au*_n_*S*_m_*] ranging from 6000 Da to 2500 Da were conserved upon native digestion (Figure S3c) and quenched upon disulfide cleavage by TCEP (Figure S3d). Signals < 2500 Da were mainly due to peptide digests since they were also detected from the native digested BSA solution (Figure S3e) and could not be quenched by TCEP. This indicated that the native digestion destroyed small clusters (<2500 Da) and cleaved out the non-core portion, but conserved the core-portion and the FRET environment of large clusters. Our data is the first to show that disulfide groups are critical for stabilizing the core structure of red BSA-AuNCs.

The core and non-core fractions were separated using a 10 kDa-cutoff membrane (Scheme 1). As shown, the core (top) fraction exhibiting red fluorescence (photo shown in Scheme 1) had nearly a 100% yield by cutoff, as the fluorescence intensity at 650 nm excited at either 295 or 350 nm was not changed by cutoff (Figure 3a). In contrast, the non-core (bottom) fraction had no detectable fluorescence (photo shown in Scheme 1 and Figure 3a). Similarly, the [Au*_n_*S*_m_*] ions in the low-mass region were also detected in the core fraction (Figure 3b) but not detected in the non-core fraction (Figure 3c). Instead, the BSA peptide digests in the mass region < 2500 Da were released to the non-core fraction and detected (Figure 3c), indicating that the non-core portion of BSA-AuNCs was digested into peptides and removed from the core portion by the 10 kDa-cutoff membrane. The core and non-core fractions were respectively denatured and alkylated, followed by trypsin digestion and subjected to nanoLC-MS^2^ analysis (Scheme 1). The fluorescence (Scheme 1 and Figure 3a) and [Au*_n_*S*_m_*] (Figure 3d) signals of the core fraction were completely quenched upon denaturation and alkylation.

**Figure 4:**
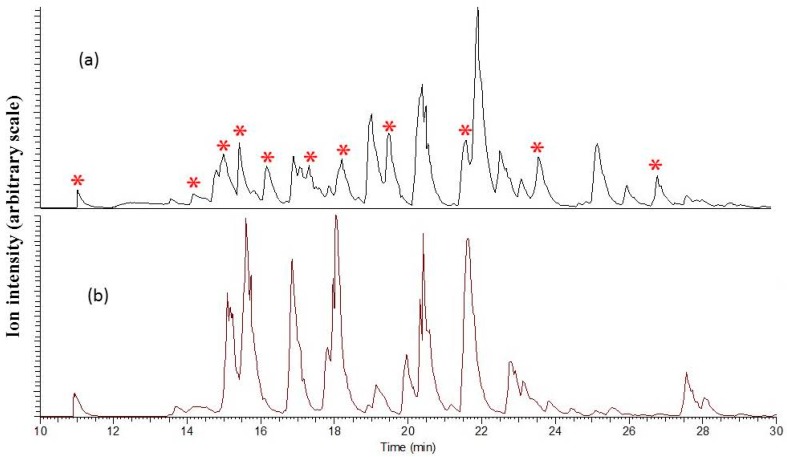
Base peak chromatogram of (**a**) the core fraction and (**b**) the non-core fraction. The star (*) indicates peaks belonging to peptides containing a non-oxidized cysteine residue. Non-labeled peaks belong to peptides containing no cysteine residue.

**Figure 5:**
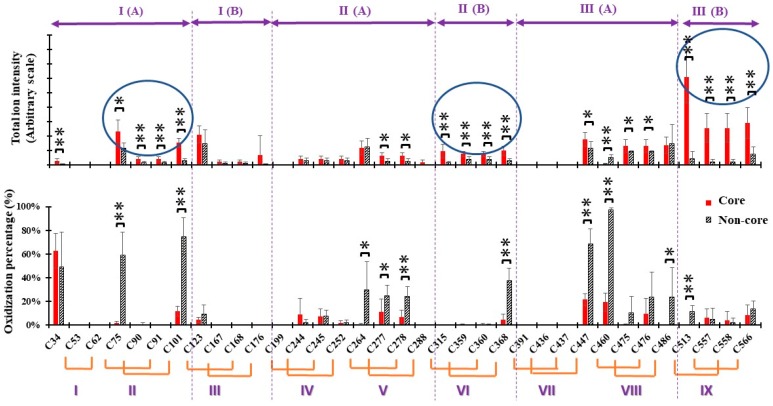
(**a**) Ion intensity and (**b**) oxidization percentage of cysteine-containing peptides covering individual cysteine sites detected from the core (red) and non-core (gray) fraction. Each data point was the average of 5–6 independent measurements with ±1 standard deviation (error bar). Differences between the core and non-core fraction were considered significant at *P* < 0.05 (*) or *P* < 0.01 (**).

Base peak chromatograms (BPC) constructed by the strongest peptides at each elution time (Figure 4a) indicated that the core fraction mainly consisted of peptides containing non-oxidized cysteine residues. In contrast, the non-core fraction mainly consisted of peptides which do not have cysteine residues (Figure 4b). This indicated that the core ligands were mainly sequences covering intact disulfide bonds of BSA. The ion intensity (Figure 5a) and oxidization percentage (Figure 5b) of peptides corresponding to each cysteine site in the core and non-core fraction were compared. Based on five to six independent experiments, a majority of cysteine sites were determined to be significantly (*P* < 0.05 or 0.01) enriched in the core fraction (Figure 5a) with a significantly (*P* < 0.05 or 0.01) lower oxidization percentage (Figure 5b) than that in the non-core fraction. This again confirmed that the peptide sequences covering the intact disulfide bonds were core ligands of BSA-AuNCs. Among them, the disulfide pair II (C75−C91/C90−C101) in domain IA, disulfide pair VI (C315−C360/C359−C368) in domain IIB, and disulfide pair IX (C513−C558/C557−C566) in domain IIIB (circled) all had the four cysteines (two disulfide groups) significantly (*P* < 0.05 or 0.01) enriched in the core fraction. Moreover, these three disulfide pairs have low global oxidization levels (< 30%) (Figure 2c), suggesting they were capping ligands. This is consistent with the –S–Au–S– “staples” motif which was generally believed to cap Au nanoclusters in a protein template [28].

According to MD simulations [33], gold clusters grow close to a number of cysteine sites across all three domains of BSA, consistent with our results that multiple cysteines were enriched in the core fraction (Figure 5a) with low (<20% except C34) oxidization percentage (Figure 5b). Additionally, the domain IIB and domain IA were reported to accommodate large clusters comprising more than 12 atoms [33], which is consistent with our results that disulfide cluster II in domain IA and disulfide cluster VI in domain IIB were significantly enriched in the core fraction (Figure 5a). Moreover, the domain IIB was further suggested to host the largest Au clusters, because a reasonable FRET separation of around 29.7 Å between W213 and gold nanoclusters located in C315 was revealed by the MD simulation [33]. Based on our data, the disulfide cluster VI may have bigger clusters than those located in the disulfide cluster II, since C101 belonging to the disulfide cluster II appeared to have a relatively high (next to C123) global oxidization percentage (Figure 2c), suggesting less intact disulfide pairs to host the large clusters. However, MD simulation did not indicate the disulfide cluster IX hosts large clusters, and further research is required to confirm this.

### 2.5. Identification of C34 as Au^+^ Binding Sites Involved in Energy Transfer

In addition to the three disulfide pairs, there are other cysteine sites enriched in the core fraction. C34 is the only free thiol of BSA and is also the only cysteine site which has a high oxidization percentage in the core fraction (Figure 5). The binding of Au at C34 was considered by others [20,43] and was suggested to be the Au ion binding site involved in the energy transfer, rather than the location of red fluorophore [20]. This is consistent with the high oxidization ratio of C34 in the core fraction since the negatively-charged sulfinic or sulfonic groups easily bind with Au cations. Similarly, those with partial disulfides (or single cysteine) significantly enriched in the core fraction (C277, C278, C447, C475, C476) may also be binding sites of metal ion or small AuNCs. Although the FRET environment was conserved upon native digestion, the only two tryptophan residues (W134 and W213) in BSA were found to be evenly distributed in the core and non-core fractions (data not shown). Thus, in addition to tryptophan, it is very likely that Au^+^ or small AuNCs located near the large AuNCs were also involved in cascaded energy transfer to further enhance red fluorescence.

According to the crystal structure of the N-isoform of BSA (PDB code: 3V03), the three disulfide pairs (II, VI, and IX) identified to host large clusters have the greatest (>40 Å^2^) accessible surface area among all cysteine sites (Supporting information Figure S5). In contrast, the largely oxidized cysteine sites (C34, C124, C264, C277, and C460) have a relatively small (<20 Å^2^) accessible surface area (Figure S5). Thus, we hypothesized that under neutral pH, AuCl_4_^−^ ions were introduced in random locations at lysine or arginine sites of the protein surface and migrated to the most accessible disulfide pairs (II, VI, and IX). Although the secondary structure was not much altered by the conformational change from N- to A-isoform, disulfide cleavages increased with such conformational changes [44,45]. This process accompanied with extensive cysteine oxidation (into cysteinesulfinic or cysteic acid), provided reducing power and spaces for subsequent growth of AuNCs at neighboring intact disulfide pair sites. This is consistent with the report [33] that when the disulfide bonds were broken during the conformational change, the propensity for the gold cluster close to cysteine residues was seen to increase.

## 3. Materials and Methods

### 3.1. Chemicals and Reagents

Bovine serum albumin (BSA), sinapinic acid (SA), 2-cyano-3-(4-hydroxyphenyl)acrylic acid (CHCA), ammonium bicarbonate, DL-dithiothreitol (DTT), Iodoacetamide (IAM), hydrochloric acid (HCl), and guanidine hydrochloride (Gn-HCl) were from Sigma-Aldrich Chemical Co (St. Louis, Mo, USA). Sodium hydroxide and ethanol were from J.T.Backer Chemical Co (Philipsburg, NJ, USA). Trifluoroacetic acid (TFA) and tetrachloroauric acid (HAuCl_4_) were from Alfa Aesar (Heysham, Lancashire, UK). Acetonitrile (ACN) was from Merck (Frankfurter Darmstadt, Germany). Formic acid (FA) and tris(2-carboxyethyl)phosphine (TCEP) were from Thermo Fisher Scientific Inc. (Walthan, MA, USA). Water (DI-water) was purified by Milli-Q plus system (Darmstadt, DE, USA) with a resistivity over 18.2 MΩ•cm.

### 3.2. Synthesis of the Red BSA-AuNC

BSA-AuNCs were synthesized at pH 12 and 37 °C (experimental details available in supporting information) following the method published previously [6,46]. A volume of 500 μL of aqueous HAuCl_4_ solution (10 mM) was added to 500 μL of BSA solution (50 mg/mL) under vigorous stirring. A volume of 50 μL of NaOH (1M) was subsequently added and the mixture was incubated at 37 °C. After 6 h, an extra 6 mM HAuCl_4_ solution was added to the mixture and the solution was incubated at 37 °C for another 12 h. The color of the solution changed from light yellow to deep brown. The solution was then dialyzed against DI-water for 12 h to remove unreacted reagents. The final solution (BSA-AuNC) was stored at 4 °C until use.

### 3.3. UV/Fluorescence Measurement

Samples were loaded onto a 400 μL quartz cuvette. Fluorescence was measured by scanning emission wavelength from 310–750 nm with excitation at 295 or 350 nm using fluorescence spectrometer (Model FS5, Edinburgh, Kirkton Campus, Livingston, UK) or photoluminescence spectrometer from Horiba (Kisshoin, Tokyo, Japan). The slit was set to be 1 nm for the excitation and 5 nm for the emission.

### 3.4. MALDI-MS Measurement

One microliter of the protein solution (~1 mg/mL) was mixed with 2 μL of the matrix which consisted of 20 mg/mL of sinapic acid (for mass range *m*/*z* 20,000–160,000) or cyano-4-hydroxycinnamic acid (for mass range m/z 500–7000) in 50% ACN containing 0.1% TFA and then applied to a standard MALDI plate. After drying, all the spectra were acquired using a MALDI-TOF MS instrument (Autoflex III MALDI-TOF, Bruker Daltonics, Billerica, MA, USA) using an accelerating voltage of 20 kV. For the high mass range, linear positive mode was scanned from 20k to 160 kDa. For the low mass range, the linear negative mode was scanned from 500 to 7000 Da.

### 3.5. X -ray Photoelectron Spectroscopy (XPS)

The dried BSA-AuNC powder was taped on a 1 × 1 cm glass and detected by XPS microprobe instrument (ESCA Model: PHI 5000 VersaProbe, Chanhassen, MN, USA). The photon binding energy from 1 to 5 nm was scanned and the acquired data were fitted for Au_4f_ peaks using CasaXPS software (Devon, UK).

### 3.6. Preparation of the Core and Non-Core Fraction of BSA-AuNC

A volume of 80 μL of the native-digested BSA-AuNC solution (10 μg/μL) was loaded onto a 10 kDa cutoff filter (Amicon, Darmstadt, Germany) and added with a buffer until a final volume of 280 μL. The membrane was then centrifuged at 10,000 rpm for 10 min. After centrifugation, the bottom cup was decanted, 200 μL DI water was added to the top cup, and then they were centrifuged again at 10,000 rpm for 10 min. This procedure was repeated six times. The top fraction was considered as the core fraction. All the collected bottom fractions were combined and considered as the non-core fraction. Both the top and bottom fractions were adjusted to a final volume of 400 μL for fluorescence or MS measurements. For LC-MS^2^ analysis, each fraction was digested again following the procedure described below.

### 3.7. Trypsin Digestion

A volume of 50 μL of the BSA-AuNC or BSA solution (10 μg/μL) was buffer exchanged to the solvent composed of 6 M Gn-HCl dissolved in ammonia bicarbonate (50 mM) using a 3 kDa cutoff filter (Amicon, Darmstadt, Germany). For denatured digestion (Scheme 1), the solution was reduced by DTT (0.25 M, pH 8) or TCEP (5 mg/mL, pH 6.8) at 37 °C for 1 h and alkylated by IAM (0.25 M) in the dark for 30 min with occasional mixings. For native digestion (Scheme 1), the solution was buffer exchanged to ammonia bicarbonate buffer (50 mM, pH 6.8) without the treatment with DTT (or TCEP)/IAM. A volume of 2 μL of trypsin (1 μg/μL) was added to both solution and the solution was incubated at 37 °C for 18 h. After digestion, the solution was dried or stored at 4 °C until analyses.

### 3.8. LC-MS^2^ Analysis

A volume of 1 μL of the sample was injected onto Waters nanoACQUITY UPLC system equipped with a precolumn (Waters, 0.180 mm × 20 mm, 5 μm C18) (Milford, MA, USA) followed by a nanocolumn (Waters, 75 μm × 25 cm, 1.7 μm C18, BEH130) at 40 °C in series coupled online to an LTQ-Orbitrap XL mass spectrometer (Thermo Fisher Scientific, San Jose, CA, USA). The sample was eluted by mobile phase A composed of 0.1% FA in DI-water and mobile phase B composed of 100% ACN in 0.1% FA using the following gradient: 0–5 min 5% B; 5–40 min 5–35% B; 40–45 min 35–90% B, 45–50 min 90% B; 50–55 min 90–5% B; 55–70 min 5% B. MS data were acquired using the data-dependent mode where one full scan with *m*/*z* 300–2000 in the Orbitrap (R = 60,000 at *m*/*z* 400) at a scan rate of 30 ms/scan was followed by the five most intense peaks for fragmentation with a normalized collision energy value of 35% in the LTQ. A repeat duration of 30 s was applied to exclude the same *m*/*z* ions from being re-selected for fragmentation.

The spectra generated in the CID-MS2 step were searched against the spectra of theoretical fragmentations (b and y ions) of the BSA sequence with a mass tolerance of ≤10 ppm of precursor ions and ± 0.8 Da of product ions with lysine specificity and two allowable miss-cleavages. Carbamidomethyl cysteine (∆m 57.0214 Da) (if the sample was alkylated) and oxidation (O, O2, O3) (∆m 15.9949 Da, ∆m 31.9898 Da, ∆m 47.9847 Da) on methionine, cysteine, histidine, tyrosine, and tryptophan were used as variable modifications. Mascot probability score (>95% confidence) was used as the filter and only the ‘rank 1′ sequence was chosen for the assignment of each MS-MS spectrum. For peptides with two miscleavages, their assigned sequences were further confirmed by manual inspection to match all highly abundant product ions. Some expected but un-identified sequences were manually extracted and confirmed by CID-MS2 spectra. The intensity of each identified peptide sequence was exported from Mascot to Excel (Microsoft Excel, Redmond, WA, USA) sheet. The oxidization percentage of a specific site was then calculated by dividing the peak intensity of all oxidized peptide sequences covering the same oxidization site by the sum of peak intensity of the corresponding un-oxidized and oxidized peptides.

### 3.9. Solvent Accessible Area

The crystal structure of the N-isoform of BSA (PDB code: 3V03) was used to estimate the solvent-accessible area via the free calculation tool in the website [47].

### 3.10. Statistics

All data were analyzed using Excel and are presented as means ± SE. Results were compared by one-way analysis of variance (ANOVA). Differences were considered significant at *P* < 0.05 or *P* < 0.01.

## 4. Conclusions

Based on a workflow using several techniques relevant to protein MS, we identified key methionine (M445, M547) and cysteine (C34, C123, C264, C277, C460) residues of BSA responsible for Au^+^ reduction and the growth of AuNCs. Moreover, we identified the disulfide pair sites C75−C91/C90−C101, C315−C360/C359−C368, and C513−C558/C557−C566 as potential nucleation sites of red AuNCs. We have also identified some other core-enriched cysteine binding sites for metal ion (C34) or small AuNCs, which may be responsible for FRET in addition to tryptophan. This is the first report on precise identification of the reducing and capping sites of BSA-AuNCs. Such site-specific information can increase the accuracy of molecular simulation and enhance the basic understanding of BSA-AuNCs. Such information can also assist in developing more precise and targeted synthesis methods to yield more homogenous BSA-AuNCs for various applications. Moreover, the reported workflow can be generally applied for studying other protein-encapsulated metal clusters.

## Supplementary Materials

The following are available online. Figure S1: XPS of the red BSA-AuNC solution; Figure S2: MALDI-MS spectra of the BSA, red BSA-AuNCs, and denature-digested BSA-AuNC solution; Figure S3: Fluorescence of the red BSA-AuNCs, native-digested BSA-AuNCs, and TCEP-added/native-digested BSA-AuNCs excited at (a) 295 nm or (b) 350 nm. [Au*_N_*S*_M_*] signals of MALDI-MS detected from (c) native-digested BSA-AuNC solution and (d) TCEP-added/native-digested BSA-AuNC solution, as well (e) BSA solution; Figure S4: LC-MS^2^ spectra of tryptic peptides containing the oxidization site on (a) C34 (b) C123 (c) 264 (d) 277 (e) C460 (f) M445 (g) M547; Figure S5: Ion intensity of W134- or W213-containing peptides (sequences shown in the inset Table) detected from the core (red) and the non-core (gray) fraction. Each data point was the average of 5-6 independent measurements with ±1 standard deviation (error bar); Figure S6: Molecular model of BSA and accessible surface area of cysteine sites; Table S1: Peptide sequences of BSA identified from BSA-AuNC solution by LC-MS^2^.
