# Supplementary material for: Identifying Reducing and Capping Sites of Protein-Encapsulated Gold Nanoclusters
Source: Molecules. 2019 Apr 25;24(8):1630. doi: 10.3390/molecules24081630 (PMC6514900; doi:10.3390/molecules24081630)
Supplement: Supplementary file 1 [file molecules-24-01630-s001.pdf]

Supporting Information

Identifying Reducing and Capping Sites of Protein-Encapsulated Gold Nanoclusters Using Proteomics

Yu-Chen Hsu (f83712@gmail.com),[1] Mei-Jou Hung (mr.comicchild@gmail.com), [1] Yi-An Chen (jelly601095@gmail.com), [1] Tsu-Fan Wang(tfwang@mail.ncyu.edu.tw), [2] Ying-ru Ou(r910061@gmail.com),[1] and Shu-Hui Chen[1]*

[1] Department of Chemistry, National Cheng Kung University, no.1 College Road, Tainan 70107, Taiwan;

[2] Department of Applied Chemistry, National Chia-Yi University, no.300 Syuefu Road, Chiayi City 60004, Taiwan

*e-mail: shchen@mail.ncku.edu.tw

Figure S1 XPS of the red BSA-AuNC solution.

Figure S2 MALDI-MS spectra of the BSA, red BSA-AuNCs, and denature-digested BSA-AuNC solution.

Figure S3 Fluorescence of the red BSA-AuNCs, native-digested BSA-AuNCs, and TCEP-added/native-digested BSA-AuNCs excited at (a) 295 nm or (b) 350 nm. [Au_NS_M] signals of MALDI-MS detected from (c) native-digested BSA-AuNC solution and (d) TCEP-added/native-digested BSA-AuNC solution, as well (e) BSA solution.

Figure S4 LC-MS^2 spectra of tryptic peptides containing the oxidization site on (a) C34 (b) C123 (c) 264 (d) 277 (e) C460 (f) M445 (g) M547.

Figure S5 Ion intensity of W134- or W213-containing peptides (sequences shown in the inset Table) detected from the core (red) and the non-core (gray) fraction. Each data point was the average of 5-6 independent measurements with ±1 standard deviation (error bar).

Figure S6 Molecular model of BSA and accessible surface area of cysteine sites

Table S1 Peptide sequences of BSA identified from BSA-AuNC solution by LC-MS^2

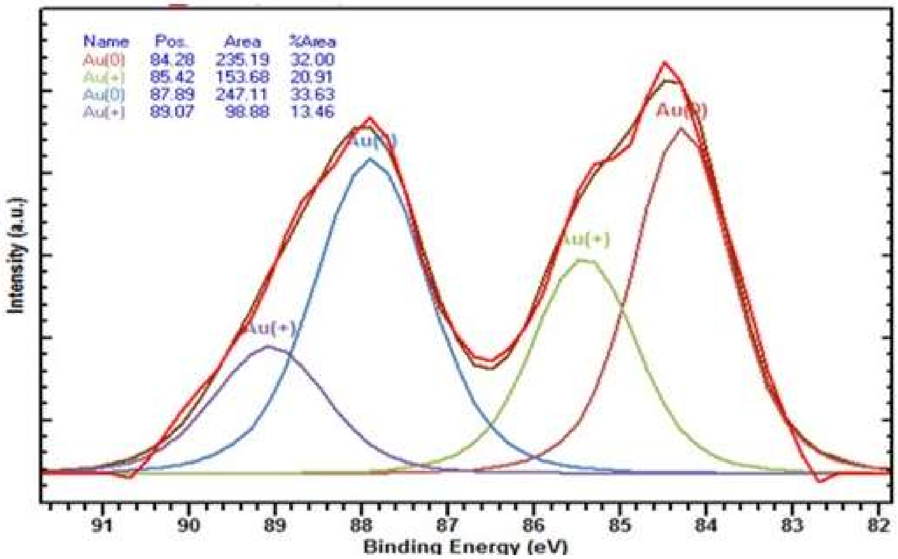

Au(0) %	Au(+) %	4f 7/2 (eV)	4f 5/2 (eV)
**65~70%**	**30~35%**	**~84.28**	**~87.89**

Figure S1 XPS of the red BSA-AuNCs solution.

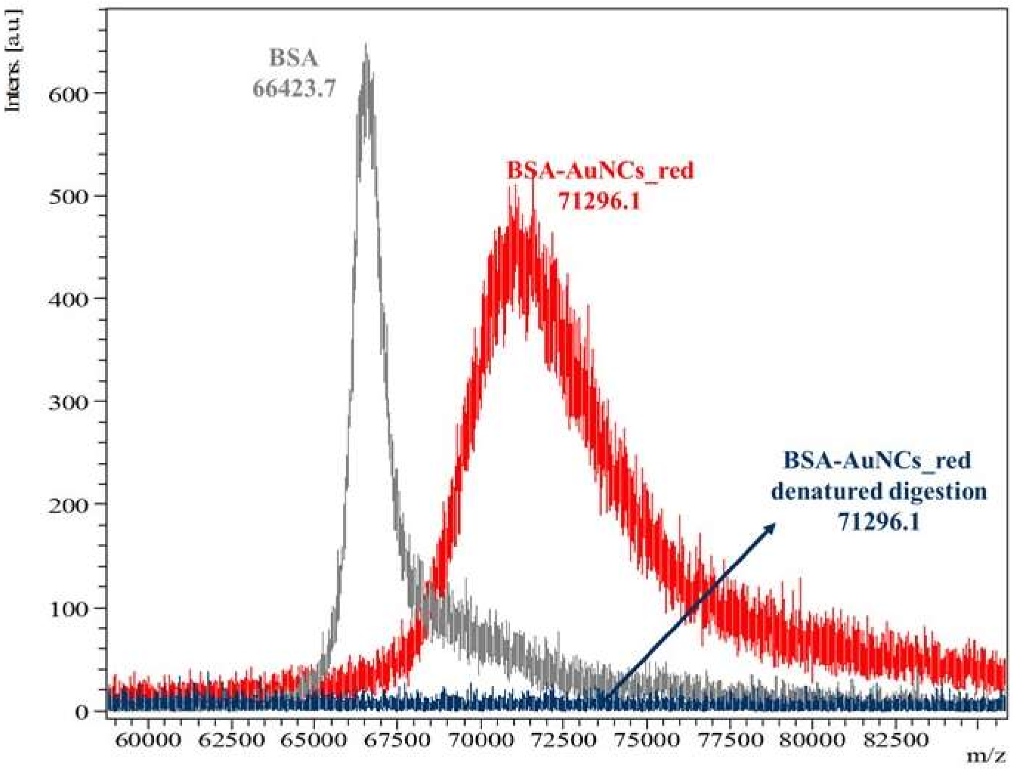

Figure S2 MALDI-MS spectra of the BSA, red BSA-AuNCs, and denature-digested BSA-AuNCs solution.

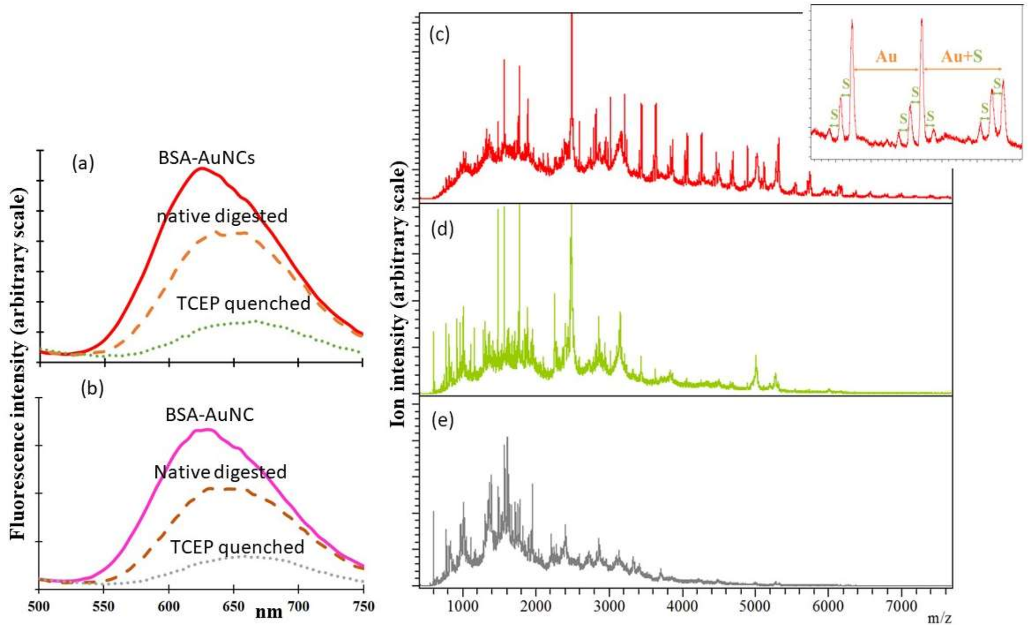

Figure S3 Fluorescence of the red BSA-AuNCs, native-digested BSA-AuNCs, and TCEP-added/native-digested BSA-AuNCs excited at (a) 295 nm or (b) 350 nm. [Au_NS_M] signals of MALDI-MS detected from (c) native-digested BSA-AuNC solution and (d) TCEP-added/native-digested BSA-AuNC solution, as well (e) native-digested BSA solution.

(a)

pep_seq	m/z (z)	intensity	RT	start	end	PTM	Site	score
GLVLIAFSQYLQQCPFDEHVK	1242.1239 (2)	383060	46.97	45	65	O_3	**C34**	92.01

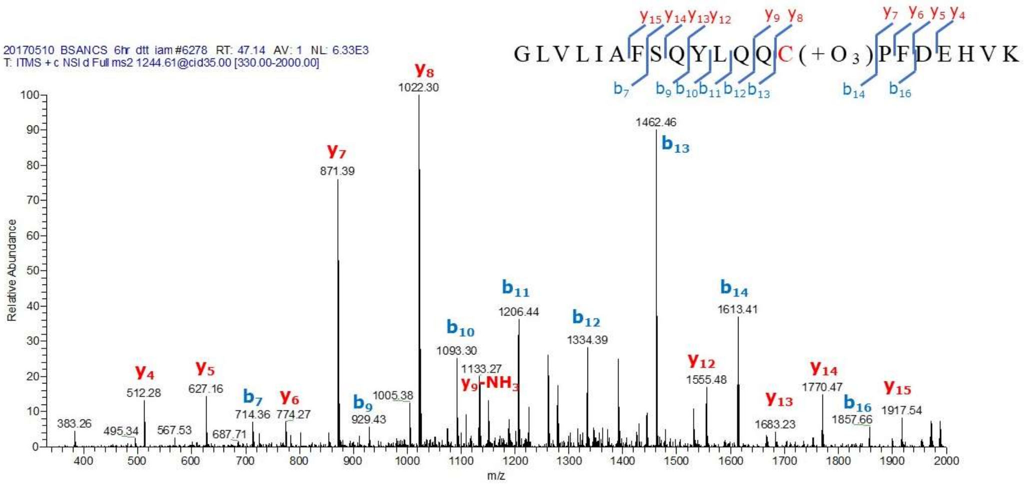

(b)

pep_seq	m/z (z)	intensity	RT	start	end	PTM	Site	score
LKPDPNTLCDEFK	776.3722 (2)	1032285	29.64	139	151	O_2	**C123**	43.41

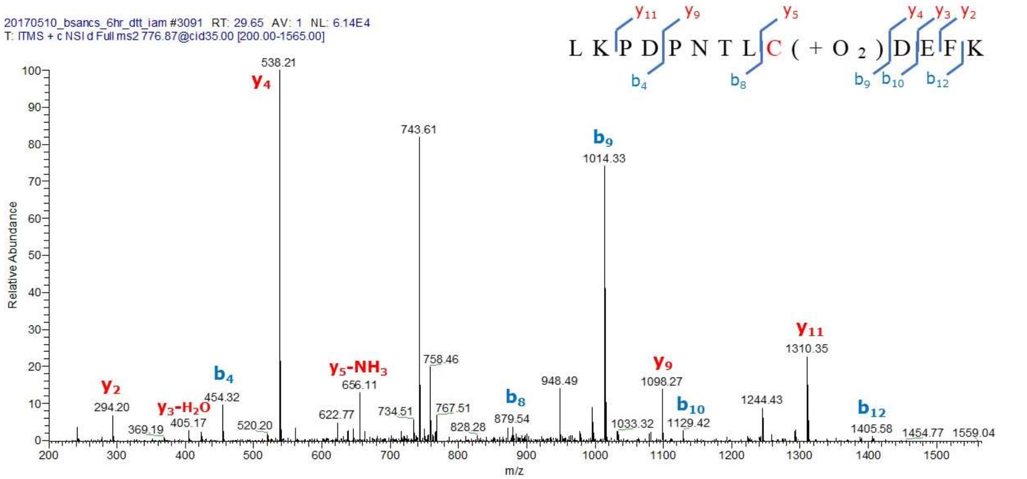

(c)

pep_seq	m/z (z)	intensity	RT	start	end	PTM	Site	score
YICDNQDTISSK	717.8079 (2)	1182700	23.81	286	297	O_3	**C264**	65.34

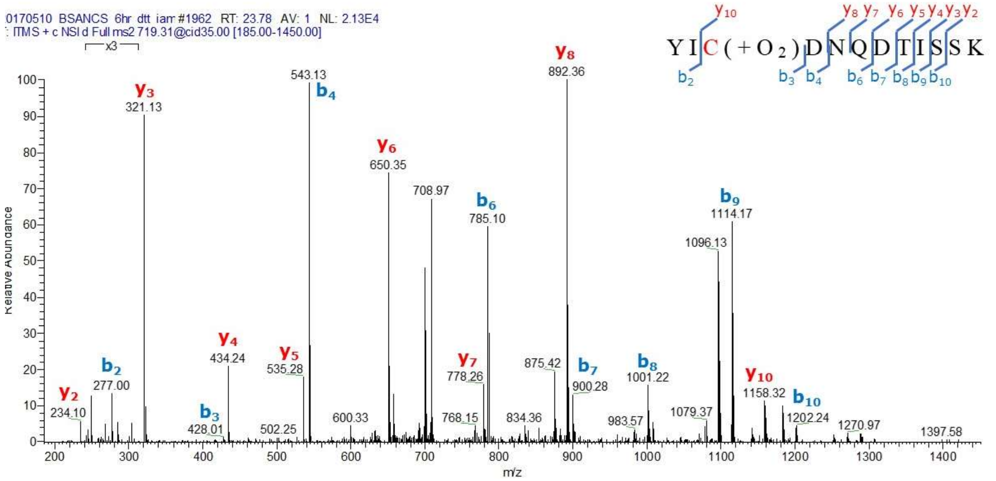

(d)

pep_seq	m/z (z)	intensity	RT	start	end	PTM	Site	score
LKECCDKPLLEK	733.8669 (2)	156729	24.88	298	309	O_3	**C277**	47.22

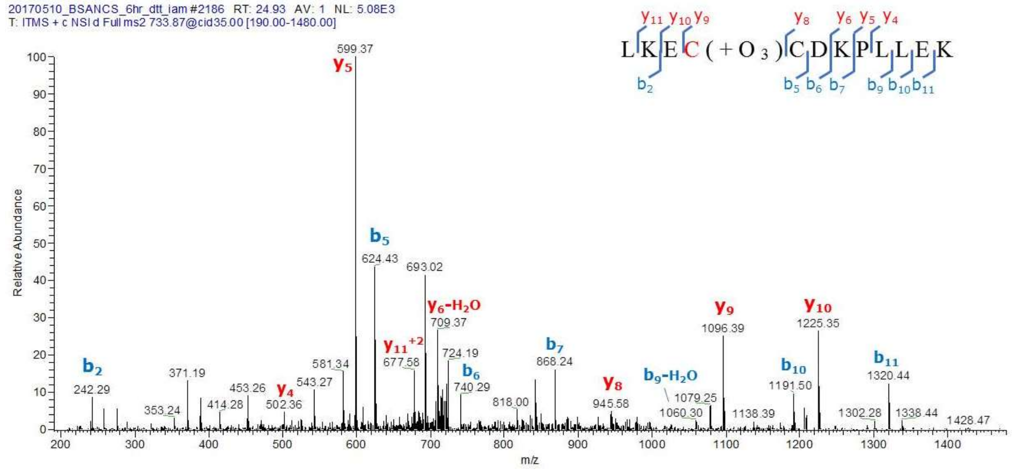

(e)

pep_seq	m/z (z)	intensity	RT	start	end	PTM	Site	score
LCVLHEK	445.226 (2)	1414303	25.84	483	489	O_3	**C460**	42.75

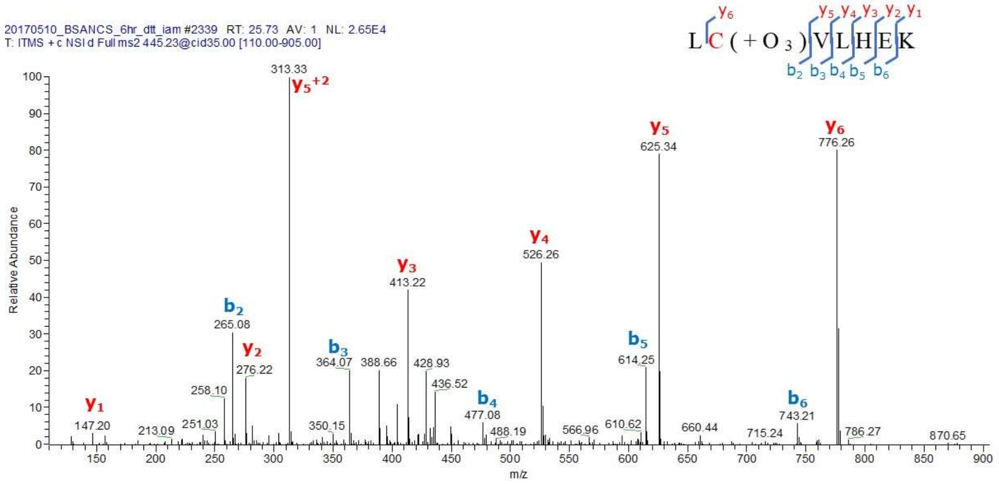

(f)

pep_seq	m/z (z)	intensity	RT	start	end	PTM	Site	score
MPCTEDYLSLILNR	842.4084 (2)	593015	42.58	469	482	O	**M445**	87.66

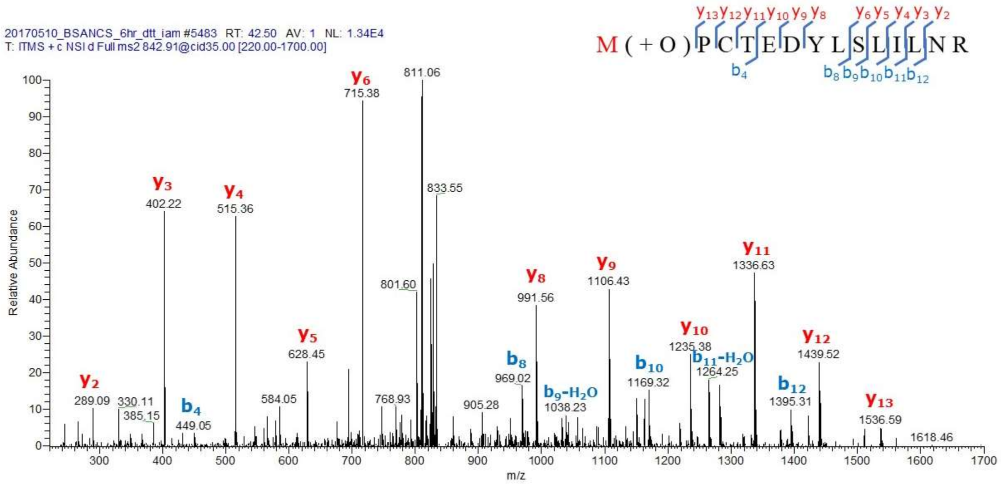

(g)

pep_seq	m/z (z)	intensity	RT	start	end	PTM	Site	score
TVMENFVAFVDK	708.347 (2)	2855674	35.87	569	580	O	**M547**	67.78

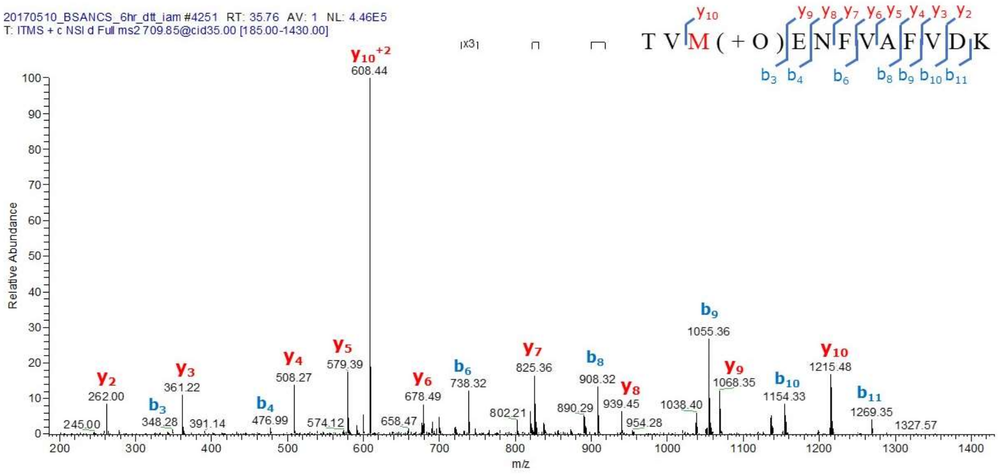

Figure S4 LC-MS^2 spectra of tryptic peptides containing the oxidization site on (a) C34 (b) C123 (c) 264 (d) 277 (e) C460 (f) M445 (g) M547.

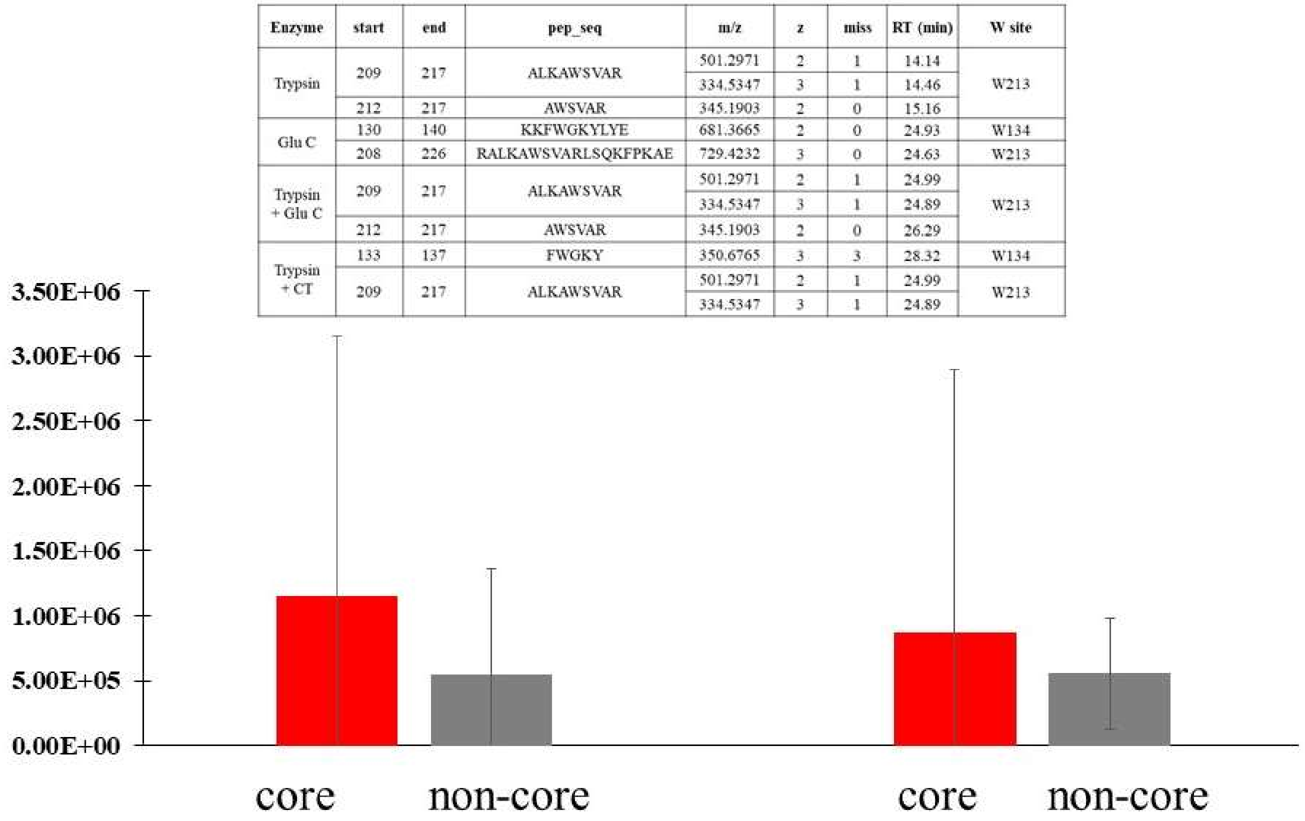

Enzyme	start	end	pep_seq	m/z	z	miss	RT (min)	W site
Trypsin	209	217	ALKAWSVAR	501.2971	2	1	14.14	W213
				334.5347	3	1	14.46	
	212	217	AWSVAR	345.1903	2	0	15.16	
Glu C	130	140	KKFWGKYLYE	681.3665	2	0	24.93	W134
	208	226	RALKAWSVARLSQKFPKAE	729.4232	3	0	24.63	W213
Trypsin + Glu C	209	217	ALKAWSVAR	501.2971	2	1	24.99	W213
				334.5347	3	1	24.89	
	212	217	AWSVAR	345.1903	2	0	26.29	
Trypsin + CT	133	137	FWGKY	350.6765	3	3	28.32	W134
	209	217	ALKAWSVAR	501.2971	2	1	24.99	W213
				334.5347	3	1	24.89	

Figure S5 Ion intensity of W134- or W213-containing peptides (sequences shown in the inset Table) detected from the core (red) and the non-core (gray) fraction. Each data point was the average of 5-6 independent measurements with ±1 standard deviation (error bar).

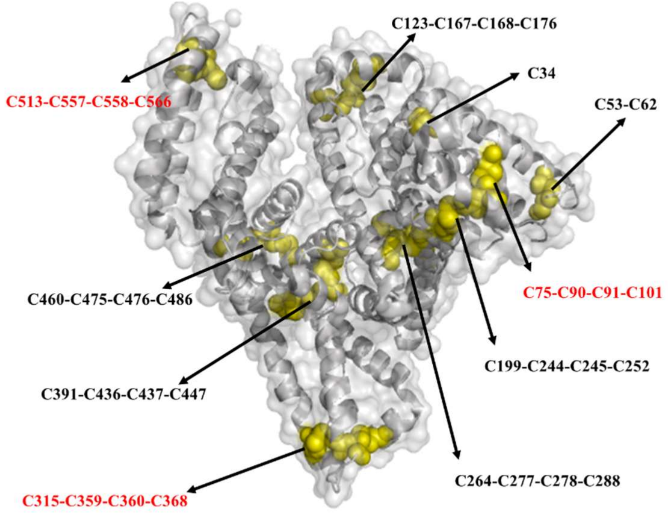

Accessible Surface Area

site	Area(Å^2)
C34	11.122
C53	0
C62	0.454
C75	39.42
C90	0
C91	40.831
C101	19.345
C123	12.673
C167	0.113
C168	34.353
C176	33.584
C199	4.618
C244	7.702
C245	5.693
C252	0
C264	18.264
C277	2.904
C278	20.33
C288	29.308

C315	24.844
C359	7.243
C360	39.823
C368	58.704
C391	12.918
C436	0
C437	23.079
C447	18.072
C460	6.708
C475	1.211
C476	10.647
C486	30.922
C513	23.646
C557	0
C558	44.605
C566	18.786

Figure S6 Molecular model of BSA and accessible surface area of cysteine sites

Table S1 Peptide sequences of BSA identified from the BSA-AuNC solution

start	end	pep_seq	modification site	pep_miss	m/z (z)	RT (min)	exp_mass	theo_mass	delta Da (exp-theo)	pep score
1	10	DTHKSEIAHR		1	398.5386(3)	3.66	1192.594	1192.5949	-0.0009	28.75
5	10	SEIAHR		0	356.6904(2)	3.69	711.3662	711.3664	-0.0002	33.38
11	20	FKDLGEEHFK		1	313.1614(4)	23.36	1248.6165	1248.6139	0.0026	27.73
		FKDLGEEHFK		1	417.2123(3)	23.7	1248.6152	1248.6139	0.0013	59.29
		FKDLGEEHFK		1	625.3152(2)	23.74	1248.6158	1248.6139	0.0019	55.06
13	20	DLGEEHFK		0	487.7323(2)	21.8	973.45	973.4505	-0.0005	42.76
21	41	GLVLIAFSQYLQQCPFDEHVK	C34(O2)	0	1234.1239(2)	46.38	2466.2333	2466.2253	0.0079	46.88
			C34(O3)	0	1242.1227(2)	45.28	2482.2308	2482.2202	0.0106	107.01
			C34(IAM)	0	1246.6401(2)	45.03	2491.2657	2491.257	0.0087	106.57
			C34(O3)	0	621.5623(4)	46.96	2482.2202	2482.2202	-0.0001	77.61
			C34(IAM)	0	623.8218(4)	45.34	2491.258	2491.257	0.001	55.1
				0	812.4236(3)	47.1	2434.2489	2434.2355	0.0134	39.38
			C34(O2)	0	823.0839(3)	44.52	2466.2298	2466.2253	0.0044	53.17
			Y30(O) ; C34(O2)	0	828.4112(3)	53.89	2482.2118	2482.2202	-0.0085	27.7
			C34(O3)	0	828.4161(3)	51.03	2482.2266	2482.2202	0.0063	70.05
			C34(IAM)	0	831.4301(3)	43.12	2491.2683	2491.257	0.0114	77.75
			Y30(O2) ; C34(O2)	0	833.7456(3)	48.57	2498.215	2498.2151	-0.0002	31.58
21	51	GLVLIAFSQYLQQCPFDEHVKLVNELTEFAK	Y30(O2)	1	1204.6191(3)	49.6	3610.8356	3610.8381	-0.0025	27.53
42	51	LVNELTEFAK		0	582.3193(2)	3.18	1162.6241	1162.6234	0.0008	56.48
52	64	TCVADESHAGCEK	C53(IAM) ; C62(IAM)	0	488.5346(3)	11.52	1462.5819	1462.5817	0.0002	40.96
			C53(O2) ; C62(IAM)	0	719.7834(2)	12.45	1437.5523	1437.55	0.0023	45.83
			C53(IAM) ; C62(O3)	0	727.7814(2)	12.51	1453.5482	1453.545	0.0032	51.94
			C53(IAM) ; C62(IAM)	0	732.3(2)	11.26	1462.5855	1462.5817	0.0039	62.89
52	76	TCVADESHAGCEKSLHTLFGDELCK	C53(IAM) ; C62(O2)	1	947.0809(3)	32.13	2838.2208	2838.2259	-0.0051	22.96
65	76	SLHTLFGDELCK		0	454.8955(3)	34.64	1361.6647	1361.6649	-0.0002	44.47
			C75(O2)	0	465.5587(3)	35.12	1393.6544	1393.6548	-0.0003	23.54
			C75(O3)	0	470.8897(3)	35.2	1409.6474	1409.6497	-0.0023	44.96
			C75(IAM)	0	473.9022(3)	32.6	1418.6847	1418.6864	-0.0017	42.49
				0	681.8398(2)	34.61	1361.665	1361.6649	0.0001	56.27
			C75(O2)	0	697.8344(2)	35.37	1393.6542	1393.6548	-0.0006	61.15
			C75(IAM)	0	710.3506(2)	33.16	1418.6866	1418.6864	0.0002	55.92
65	81	SLHTLFGDELCKVASLR	C75(O3)	1	485.0001(4)	35.54	1935.9713	1935.9724	-0.0011	47.87
			C75(IAM)	1	487.2589(4)	38.47	1945.0067	1945.0091	-0.0025	32.21
			C75(O2)	1	640.9991(3)	38.51	1919.9754	1919.9775	-0.0021	34.29
			C75(O3)	1	646.3322(3)	35.38	1935.9746	1935.9724	0.0022	45.67
			C75(IAM)	1	649.3447(3)	38.38	1945.0122	1945.0091	0.003	43.2
			C75(O2)	1	960.9985(2)	38.62	1919.9825	1919.9775	0.005	44.78
82	93	ETYGDMADCCEK	C91(IAM)	0	711.2555(2)	24.57	1420.4964	1420.4945	0.0019	60.82
			M87(O) ; C90(IAM)	0	719.2538(2)	18.85	1436.4931	1436.4894	0.0037	34.22
			C90(IAM) ; C91(O2)	0	727.2493(2)	22.99	1452.484	1452.4843	-0.0003	44.54
			C90(IAM) ; C91(IAM)	0	739.7646(2)	21.98	1477.5146	1477.516	-0.0013	74.15
			M87(O) ; C90(IAM) ; C91(IAM)	0	747.7636(2)	20.48	1493.5127	1493.5109	0.0018	56.61
82	98	ETYGDMADCCEKQEPER	M87(O2) ; C91(O3)	1	1042.3838(2)	20.04	2082.753	2082.7452	0.0078	20.83
			M87(O2) ; C90(O) ; C91(O2)	1	1042.3846(2)	20.31	2082.7547	2082.7452	0.0095	23.68
			M87(O2) ; C90(O3) ; C91(O)	1	1050.3792(2)	20.43	2098.7437	2098.7401	0.0036	48.47
			M87(O2) ; C90(O2) ; C91(O)	1	695.2561(3)	19.96	2082.7465	2082.7452	0.0013	21.42
94	98	QEPER		0	329.6615(2)	3.62	657.3084	657.3082	0.0002	27.43
99	106	NECFLSHK	C101(O3)	0	513.2213(2)	25.09	1024.428	1024.4284	-0.0004	33.27
			C101(IAM)	0	517.7396(2)	20.88	1033.4647	1033.4651	-0.0004	32.35
99	114	NECFLSHKDDSPDLPK		1	615.6205(3)	27.18	1843.8396	1843.841	-0.0014	41.03
			C101(O3)	1	631.6158(3)	28.94	1891.8257	1891.8258	-0.0001	46.49
			C101(IAM)	1	634.6279(3)	26.17	1900.862	1900.8625	-0.0006	37.97
			C101(O2)	1	938.9262(2)	28.4	1875.8379	1875.8309	0.007	41.83
			C101(O3)	1	946.9219(2)	28.97	1891.8293	1891.8258	0.0035	53.79
			C101(IAM)	1	951.4421(2)	26.12	1900.8697	1900.8625	0.0072	77.06
107	114	DDSPDLPK		0	443.7112(2)	21.91	885.4079	885.408	-0.0001	47.2
115	127	LKPDPNTLCDEFK		0	507.2529(3)	32.03	1518.7368	1518.7388	-0.002	43.51
			C123(O2)	0	517.9161(3)	29.69	1550.7264	1550.7286	-0.0022	28.82
			C123(IAM)	0	526.261(3)	29.64	1575.7613	1575.7603	0.001	36.35
				0	760.3776(2)	32.09	1518.7406	1518.7388	0.0018	40.64
			C123(O2)	0	776.3716(2)	29.41	1550.7287	1550.7286	0.0001	46.95
			C123(O3)	0	784.3694(2)	29.48	1566.7242	1566.7236	0.0007	47.48
			C123(IAM)	0	788.8887(2)	29.68	1575.7628	1575.7603	0.0025	45.33
115	131	LKPDPNTLCDEFKADEK	C123(O3)	1	1005.973(2)	28.19	2009.9315	2009.9252	0.0063	47.65
			C123(O2)	1	665.6519(3)	28.56	1993.9337	1993.9302	0.0035	22.74
			C123(O3)	1	670.9825(3)	27.93	2009.9256	2009.9252	0.0005	38.17
			C123(IAM)	1	673.9941(3)	29.1	2018.9606	2018.9619	-0.0013	22.16
			C123(O2)	1	997.9749(2)	28.3	1993.9352	1993.9302	0.0049	29.28

start	end	pep_seq	modification site	pep_miss	m/z (z)	RT (min)	exp_mass	theo_mass	delta Da (exp-theo)	pep score
132	136	KFWGK		1	333.1923(2)	20.73	664.37	664.3697	0.0003	31.49
133	143	FWGKYLYEIAR		1	482.5901(3)	36.86	1444.7486	1444.7503	-0.0017	28.25
137	143	YLYEIAR		0	464.2501(2)	29.14	926.4856	926.4861	-0.0006	37.74
144	159	RHPYFYAPELLYYANK		1	1023.0189(2)	35.93	2044.0232	2044.0206	0.0025	76.67
				1	512.0117(4)	35.81	2044.0175	2044.0206	-0.0031	37.89
				1	682.3475(3)	35.91	2044.0208	2044.0206	0.0001	68.93
145	159	HPYFYAPELLYYANK		0	630.3142(3)	38.7	1887.9208	1887.9195	0.0013	46.79
				0	944.9695(2)	38.41	1887.9244	1887.9195	0.0049	51.45
160	173	YNGVFQECCQAEDK	C168(IAM)	0	845.8468(2)	29.32	1689.6791	1689.6763	0.0028	73.78
			C167(IAM) ; C168(IAM)	0	874.3577(2)	26.11	1746.7009	1746.6978	0.0031	85.54
174	180	GACLLPK		0	351.2039(2)	26.44	700.3933	700.3942	-0.0008	32.09
			C176(O2)	0	367.1993(2)	28.21	732.384	732.384	0	26.26
			C176(IAM)	0	379.7148(2)	25.18	757.415	757.4156	-0.0006	42.38
181	185	IETMR		0	325.1707(2)	13.34	648.3268	648.3265	0.0003	26.91
			M184(O)	0	333.1679(2)	8.91	664.3212	664.3214	-0.0002	26.52
181	194	IETMREKVLASSAR	M184(O3)	2	819.9297(2)	0.36	1637.8448	1637.8406	0.0042	NA*
195	204	QRLRCASIQK		2	601.8486(2)	25.81	1201.6826	1201.6713	0.0113	NA*
197	204	LRCASIQK	C199(O3)	1	483.7552(2)	16.38	965.4959	965.4964	-0.0004	29.84
199	204	CASIQK	C199(IAM)	0	353.6813(2)	9.28	705.3481	705.3479	0.0002	34.59
209	217	ALKAWSVAR		1	334.5345(3)	25.61	1000.5817	1000.5818	-0.0001	27.96
				1	501.2971(2)	25.31	1000.5797	1000.5818	-0.0021	45.28
212	221	AWSVARLSQK		1	573.3247(2)	26.38	1144.6349	1144.6353	-0.0004	21.81
218	224	LSQKFPK		1	424.2552(2)	18.7	846.4958	846.4963	-0.0006	28.96
222	232	FPKAEFVEVTK		1	432.2391(3)	29.72	1293.6955	1293.6969	-0.0014	63.26
				1	647.8558(2)	29.75	1293.6971	1293.6969	0.0002	71.28
225	232	AEFVEVTK		0	461.7474(2)	25.16	921.4803	921.4807	-0.0004	45.72
225	239	AEFVEVTKLVTDLTK		1	564.9852(3)	41.62	1691.9337	1691.9346	-0.0009	68.78
				1	846.9749(2)	39.14	1691.9352	1691.9346	0.0006	105.47
233	239	LVTDLTK		0	395.2394(2)	23.99	788.4643	788.4644	-0.0001	40.04
240	256	VHKECCHGDLLECADDR	C244(O2) ; C245(O2) ; C252(O2)	1	680.268(3)	24.64	2037.7822	2037.7826	-0.0004	23.28
			C244(O3) ; C245(O) ; C252(O2)	1	680.2693(3)	24.59	2037.7862	2037.7826	0.0036	28.55
			C244(O2) ; C245(O2) ; C252(IAM)	1	688.6127(3)	24.41	2062.8164	2062.8143	0.0021	43.94
			C244(O2) ; C245(IAM) ; C252(IAM)	1	696.9567(3)	23.05	2087.8484	2087.8459	0.0024	31.51
			H241(O) ; C244(O) ; C245(IAM) ; C252(IAM)	1	696.9571(3)	22.99	2087.8494	2087.8459	0.0035	35.45
			H241(O2) ; C245(IAM) ; C252(IAM)	1	696.9571(3)	22.99	2087.8494	2087.8459	0.0035	29.42
243	256	ECCHGDLLECADDR	C244(IAM) ; C252(IAM)	0	564.8847(3)	25.9	1691.6323	1691.6338	-0.0015	36.62
			C244(IAM) ; C245(O) ; H246(O) ; C252(IAM)	0	575.548(3)	27.24	1723.6221	1723.6236	-0.0015	38.38
			C244(IAM) ; C245(O2) ; C252(IAM)	0	575.5493(3)	27.09	1723.6259	1723.6236	0.0023	32.35
			C244(IAM) ; C245(IAM) ; C252(IAM)	0	583.8928(3)	24.93	1748.6566	1748.6553	0.0014	46.56
			C244(IAM) ; C252(IAM)	0	846.8252(2)	26.09	1691.6358	1691.6338	0.002	73.19
			C244(IAM) ; C245(O2) ; C252(IAM)	0	862.8206(2)	26.8	1723.6267	1723.6236	0.0031	54.44
			C244(IAM) ; C245(IAM) ; C252(O3)	0	870.8181(2)	25.79	1739.6217	1739.6185	0.0031	53.24
			C244(IAM) ; C245(O2) ; H246(O) ; C252(IAM)	0	870.8184(2)	27.36	1739.6223	1739.6185	0.0038	65.82
			C244(IAM) ; C245(O3) ; C252(IAM)	0	870.8188(2)	27.01	1739.6231	1739.6185	0.0046	57.79
			C244(IAM) ; C245(O2) ; H246(O) ; C252(IAM)	0	870.819(2)	27.07	1739.6235	1739.6185	0.005	53.97
			C244(IAM) ; C245(IAM) ; C252(IAM)	0	875.3374(2)	25.34	1748.6603	1748.6553	0.005	93.27
262	273	YICDNQDTISSK		0	693.8154(2)	23.79	1385.6162	1385.6133	0.0029	66.75
			C264(O2)	0	709.8098(2)	22.84	1417.605	1417.6031	0.0019	60.98
			C264(O3)	0	717.8079(2)	23.78	1433.6012	1433.598	0.0031	65.34
			C264(IAM)	0	722.3275(2)	3.14	1442.6405	1442.6347	0.0057	67.76
262	275	YICDNQDTISSKLK	C264(IAM)	1	562.2784(3)	24.58	1683.8133	1683.8138	-0.0005	30.39
274	285	LKECCDKPLLEK	C277(IAM) ; C278(IAM)	1	383.9505(4)	21.33	1531.7729	1531.7738	-0.0009	32.67
				1	473.585(3)	23.77	1417.7331	1417.7309	0.0022	23.82
			C277(O2)	1	484.2466(3)	24.49	1449.7179	1449.7207	-0.0028	29.63
			C278(O3)	1	489.5785(3)	24.89	1465.7136	1465.7156	-0.002	32.48
			C277(O2) ; C278(O)	1	489.5786(3)	24.92	1465.714	1465.7156	-0.0016	28.41
			C277(O2) ; C278(O3)	1	500.242(3)	25.16	1497.7041	1497.7054	-0.0013	20.97
			C277(O2) ; C278(IAM)	1	503.2555(3)	22.79	1506.7446	1506.7422	0.0024	24.79
			C277(O3) ; C278(IAM)	1	508.5857(3)	23.33	1522.7352	1522.7371	-0.0019	37.28
			C277(IAM) ; C278(O3)	1	508.5858(3)	23.09	1522.7357	1522.7371	-0.0014	30.51
			C277(IAM) ; C278(IAM)	1	511.5985(3)	21.3	1531.7738	1531.7738	0	36.04
			C277(O2)	1	725.8683(2)	24.48	1449.7221	1449.7207	0.0015	26.26
			C277(O3)	1	733.8669(2)	24.93	1465.7193	1465.7156	0.0037	47.22
			C277(O2) ; C278(O2)	1	741.8647(2)	24.45	1481.7148	1481.7105	0.0043	38.79
			C277(O2) ; C278(O3)	1	749.8614(2)	25.14	1497.7082	1497.7054	0.0028	46.11
			C277(O2) ; C278(IAM)	1	754.3791(2)	22.75	1506.7437	1506.7422	0.0016	45.12
			C277(O3) ; C278(IAM)	1	762.3763(2)	23.08	1522.738	1522.7371	0.0009	63.06
			C277(IAM) ; C278(O3)	1	762.3768(2)	23.58	1522.7391	1522.7371	0.002	59.05
			C277(IAM) ; C278(IAM)	1	766.8956(2)	21.31	1531.7766	1531.7738	0.0028	56.67

start	end	pep_seq	modification site	pep_miss	m/z (z)	RT (min)	exp_mass	theo_mass	delta Da (exp-theo)	pep score
276	285	ECCDKPLLEK	C277(IAM) ; C278(IAM)	0	431.2051(3)	21.14	1290.5935	1290.5948	-0.0013	25
			C277(IAM) ; C278(IAM)	0	646.3045(2)	21.16	1290.5945	1290.5948	-0.0003	38.57
286	294	SHCIAEVEK	C288(IAM)	0	358.1747(3)	16.99	1071.5021	1071.5019	0.0003	33.33
			C288(O2)	0	524.2425(2)	18.87	1046.4704	1046.4702	0.0002	35.81
			C288(O3)	0	532.2398(2)	20.32	1062.4651	1062.4651	-0.0001	58.75
			H287(O2) ; C288(O)	0	532.2401(2)	20.27	1062.4656	1062.4651	0.0004	44.69
			C288(IAM)	0	536.7586(2)	17.6	1071.5027	1071.5019	0.0008	41.23
286	312	SHCIAEVEKDAIPENLPPLTADFAEDK	H287(O) ; C288(O2)	1	1000.8127(3)	39.47	2999.4162	2999.407	0.0093	26.85
			C288(IAM)	1	1003.8235(3)	36.84	3008.4488	3008.4437	0.0051	66.63
			C288(O2)	1	995.4787(3)	38.57	2983.4143	2983.412	0.0022	38.43
286	316	SHCIAEVEKDAIPENLPPLTADFAEDKDVCK	H287(O) ; C288(O) ; C315(IAM)	2	1162.8881(3)	37.14	3485.6424	3485.633	0.0093	44.35
			C288(O2) ; C315(IAM)	2	1162.8887(3)	37.2	3485.6442	3485.633	0.0112	47.6
			C288(IAM) ; C315(IAM)	2	1171.2283(3)	35.54	3510.663	3510.6647	-0.0017	77.28
			C288(IAM) ; C315(IAM)	2	703.1409(5)	35.55	3510.6683	3510.6647	0.0036	35.93
			C288(O2) ; C315(IAM)	2	872.4158(4)	37.14	3485.634	3485.633	0.001	30.16
			C288(IAM) ; C315(IAM)	2	878.6736(4)	35.61	3510.6655	3510.6647	0.0008	50.06
286	322	SHCIAEVEKDAIPENLPPLTADFAEDKDVCKNYQEAK	C288(IAM) ; C315(IAM)	3	849.8105(5)	34.63	4244.0164	4244.0041	0.0122	29.71
295	312	DAIPENLPPLTADFAEDK		0	652.6583(3)	39.02	1954.953	1954.9524	0.0006	59.68
				0	978.4866(2)	38.19	1954.9587	1954.9524	0.0064	55.68
295	316	DAIPENLPPLTADFAEDKDVCK		1	1201.0875(2)	38.04	2400.1605	2400.1519	0.0086	73.97
			C315(O3)	1	1225.0786(2)	38.45	2448.1427	2448.1366	0.0061	86.1
			C315(IAM)	1	1229.5992(2)	37.13	2457.1839	2457.1733	0.0106	80.12
				1	801.0584(3)	37.97	2400.1532	2400.1519	0.0014	26.53
			C315(O3)	1	817.0541(3)	38.6	2448.1406	2448.1366	0.004	42.89
			C315(IAM)	1	820.0667(3)	36.37	2457.1783	2457.1733	0.005	30.85
295	322	DAIPENLPPLTADFAEDKDVCKNYQEAK	C315(IAM)	2	1064.5144(3)	35.61	3190.5214	3190.5128	0.0086	44.43
317	322	NYQEAK		0	376.682(2)	3.66	751.3495	751.35	-0.0006	29.95
323	335	DAFLGSFLYEYSR		0	784.3679(2)	62.9	1566.7212	1566.7354	-0.0143	24.85
323	336	DAFLGSFLYEYSRR		1	575.2855(3)	40.08	1722.8346	1722.8365	-0.002	22.8
				1	862.4274(2)	39.82	1722.8402	1722.8365	0.0036	27.95
336	347	RHPEYAVSVLLR		1	480.6087(3)	30.29	1438.8044	1438.8045	-0.0001	85.85
				1	720.4111(2)	30.02	1438.8076	1438.8045	0.0031	49.34
337	347	HPEYAVSVLLR		0	428.575(3)	32.82	1282.7032	1282.7034	-0.0001	45.84
				0	642.36(2)	32.85	1282.7054	1282.7034	0.0021	42.72
351	362	EYEATLEECCAK	C359(IAM) ; C360(IAM)	0	501.5423(3)	24.37	1501.6052	1501.6065	-0.0013	29.2
				0	694.7888(2)	29.78	1387.5631	1387.5635	-0.0004	49.3
			C359(IAM)	0	723.3003(2)	26.71	1444.586	1444.585	0.001	61.45
			C359(IAM) ; C360(O2)	0	739.2954(2)	25.11	1476.5763	1476.5748	0.0015	50.27
			C359(O2) ; C360(IAM)	0	739.2958(2)	25.02	1476.577	1476.5748	0.0022	58.58
			C359(IAM) ; C360(IAM)	0	751.8118(2)	24.23	1501.609	1501.6065	0.0025	65.73
351	375	EYEATLEECCAKDDPHACYSTVFDK	C359(IAM)	1	975.4089(3)	32.66	2923.2048	2923.1986	0.0062	31.23
363	375	DDPHACYSTVFDK		0	499.8817(3)	29.04	1496.6232	1496.6242	-0.001	38.56
			H366(O) ; C368(O)	0	510.5451(3)	29.6	1528.6136	1528.614	-0.0005	23.39
			C368(IAM)	0	518.8902(3)	27.52	1553.6488	1553.6457	0.0031	42.99
				0	749.3206(2)	29	1496.6267	1496.6242	0.0025	45.12
			C368(O2)	0	765.3144(2)	29.93	1528.6142	1528.614	0.0002	35.36
			C368(O3)	0	773.3126(2)	30.45	1544.6107	1544.6089	0.0017	37.04
			Y369(O3)	0	773.3129(2)	30.42	1544.6113	1544.6089	0.0024	40.52
			C368(IAM)	0	777.8311(2)	26.32	1553.6476	1553.6457	0.0019	49.57
363	377	DDPHACYSTVFDKLK	C368(IAM)	1	599.2823(3)	31.23	1794.8252	1794.8247	0.0005	27.92
376	388	LKHLVDEPQNLIK		1	516.3046(3)	27.84	1545.8921	1545.8878	0.0042	38.3
				1	773.953(2)	27.66	1545.8915	1545.8878	0.0036	44.68
378	388	HLVDEPQNLIK		0	435.9101(3)	28.21	1304.7086	1304.7088	-0.0003	38
				0	653.3621(2)	28.43	1304.7097	1304.7088	0.0009	47.43
389	396	QNCDQFEK	C391(O3)	0	530.2061(2)	15.38	1058.3977	1058.3975	0.0002	20.9
			C391(IAM)	0	534.7246(2)	14.37	1067.4347	1067.4342	0.0005	31.94
397	409	LGEYGFQNALIVR		0	493.9363(3)	36.96	1478.7872	1478.7881	-0.001	76.29
				0	740.4015(2)	36.72	1478.7884	1478.7881	0.0003	88.64
397	412	LGEYGFQNALIVRYTR		1	634.0071(3)	34.68	1898.9994	1899.0003	-0.0008	55.22
410	427	YTRKVPQVSTPTLVEVSR		2	687.3885(3)	28.52	2059.1436	2059.1426	0.001	30.46
413	427	KVPQVSTPTLVEVSR		1	547.3171(3)	29	1638.9294	1638.9305	-0.0011	64.89
				1	820.4738(2)	28.92	1638.933	1638.9305	0.0025	78.53
413	431	KVPQVSTPTLVEVSRSLGK		2	675.7287(3)	30.6	2024.1643	2024.163	0.0013	55.13
414	427	VPQVSTPTLVEVSR		0	504.619(3)	32.21	1510.8352	1510.8355	-0.0003	62.28
				0	756.4255(2)	31.28	1510.8365	1510.8355	0.001	64.73
436	444	CCTKPESER	C436(IAM) ; C437(IAM)	0	583.7506(2)	3.62	1165.4867	1165.4856	0.0011	41.22

start	end	pep_seq	modification site	pep_miss	m/z (z)	RT (min)	exp_mass	theo_mass	delta Da (exp-theo)	pep score
445	458	MPCTEDYLSLILNR		0	556.6094(3)	44.13	1666.8063	1666.8058	0.0005	31.92
			M445(O)	0	561.941(3)	42.53	1682.8013	1682.8008	0.0005	42.37
			C447(O) ; Y451(O)	0	567.2724(3)	46.11	1698.7954	1698.7957	-0.0003	31.92
			C447(O2)	0	567.2725(3)	45.99	1698.7957	1698.7957	0.0001	37.46
			M445(O) ; Y451(O)	0	567.2726(3)	46.24	1698.7959	1698.7957	0.0003	35.67
			M445(O) ; Y451(O2)	0	572.6043(3)	44.54	1714.7911	1714.7906	0.0005	42.27
			M445(O) ; C447(O2)	0	572.6043(3)	44.53	1714.7911	1714.7906	0.0005	38.04
			C447(O) ; Y451(O2)	0	572.6044(3)	44.32	1714.7915	1714.7906	0.0009	29.49
			C447(IAM)	0	575.6169(3)	43.4	1723.8288	1723.8273	0.0015	47.37
			M445(O) ; Y451(O3)	0	577.9355(3)	43.27	1730.7846	1730.7855	-0.0009	43.24
			M445(O3) ; C447(O)	0	577.9355(3)	43.26	1730.7846	1730.7855	-0.0009	37.15
			M445(O) ; C447(O3)	0	577.9359(3)	45.02	1730.7857	1730.7855	0.0002	49.54
			M445(O) ; C447(IAM)	0	580.9482(3)	41.73	1739.8229	1739.8222	0.0007	41.19
				0	834.4107(2)	44.08	1666.8069	1666.8058	0.001	106.61
			M445(O)	0	842.4084(2)	42.5	1682.8023	1682.8008	0.0016	88.08
			C447(O2)	0	850.4064(2)	46.18	1698.7982	1698.7957	0.0025	49.68
			M445(O) ; C447(O2)	0	858.4035(2)	43.8	1714.7925	1714.7906	0.0019	54.68
			C447(IAM)	0	862.9222(2)	43.33	1723.8299	1723.8273	0.0026	106.27
			M445(O) ; C447(O3)	0	866.4013(2)	42.95	1730.7881	1730.7855	0.0026	91.18
			M445(O) ; C447(IAM)	0	870.9174(2)	40.76	1739.8202	1739.8222	-0.0021	78.05
			M445(O2) ; C447(O3)	0	874.4019(2)	45.7	1746.7893	1746.7804	0.0089	40.17
			M445(O2) ; C447(IAM)	0	878.9176(2)	42.41	1755.8207	1755.8171	0.0035	57.28
			M445(O3) ; C447(IAM)	0	886.905(2)	42.17	1771.7955	1771.812	-0.0165	74.94
459	465	LCVLHEK	C460(IAM)	0	300.1654(3)	21.04	897.4743	897.4742	0.0001	22.61
			C460(O3)	0	445.226(2)	25.55	888.4374	888.4375	-0.0001	43.55
			C460(IAM)	0	449.7441(2)	21.06	897.4737	897.4742	-0.0005	39.04
459	471	LCVLHEKTPVSEK	C460(IAM)	1	513.9447(3)	23.36	1538.8123	1538.8127	-0.0004	29.22
466	483	TPVSEKVTKCCTESLVNR	C476(O2)	2	676.0092(3)	22.64	2025.0056	2024.9871	0.0186	20.26
475	483	CCTESLVNR	C475(IAM)	0	541.2419(2)	22.66	1080.4692	1080.4692	0	54.29
			C475(IAM) ; C476(O2)	0	557.2363(2)	23.53	1112.458	1112.459	-0.001	21.8
			C475(O2) ; C476(IAM)	0	557.2368(2)	23.27	1112.459	1112.459	-0.0001	28.85
			C475(IAM) ; C476(O3)	0	565.2336(2)	24.17	1128.4526	1128.4539	-0.0013	42.86
			C475(IAM) ; C476(IAM)	0	569.7525(2)	21.27	1137.4905	1137.4907	-0.0002	53.63
484	499	RPCFSALTPDETYVPK		0	608.6373(3)	32.39	1822.8902	1822.8924	-0.0022	35.29
			C486(O2)	0	619.3019(3)	33.89	1854.884	1854.8822	0.0018	34.74
			C486(O3)	0	624.6331(3)	32.53	1870.8773	1870.8771	0.0002	23.33
			C486(IAM)	0	627.6454(3)	31.35	1879.9143	1879.9138	0.0005	40.87
				0	912.4553(2)	32.41	1822.8961	1822.8924	0.0037	48.64
			C486(O2)	0	928.4498(2)	33.8	1854.885	1854.8822	0.0028	45.33
			C486(O3)	0	936.4489(2)	32.87	1870.8833	1870.8771	0.0062	73.72
			C486(IAM)	0	940.9673(2)	31.62	1879.92	1879.9138	0.0062	40.12
500	504	AFDEK		0	305.1474(2)	10.71	608.2803	608.2806	-0.0003	26.83
500	520	AFDEKLFTFHADICTLPDTEK	C513(IAM)	1	833.4031(3)	37.4	2497.1876	2497.1835	0.004	36.29
505	520	LFTFHADICTLPDTEK		0	617.6385(3)	37.81	1849.8936	1849.892	0.0016	34.74
			C513(O3)	0	633.6333(3)	36.71	1897.8781	1897.8768	0.0013	41.45
			C513(IAM)	0	636.6438(3)	62.18	1906.9096	1906.9135	-0.0039	45.71
				0	925.9542(2)	37.71	1849.8939	1849.892	0.0019	92.38
			C513(O2)	0	941.9506(2)	37.26	1881.8867	1881.8819	0.0048	36.36
			C513(O3)	0	949.9472(2)	37.67	1897.8799	1897.8768	0.0031	56.2
			H509(O) ; C513(O2)	0	949.9476(2)	37.22	1897.8807	1897.8768	0.0039	42.12
			C513(IAM)	0	954.4656(2)	35.91	1906.9167	1906.9135	0.0032	65.5
524	533	KQTALVELLK		1	381.5764(3)	31.98	1141.7072	1141.707	0.0002	57.47
				1	571.8611(2)	52.18	1141.7077	1141.707	0.0007	52.02
525	533	QTALVELLK		0	507.8139(2)	35.99	1013.6133	1013.6121	0.0012	61.51
525	537	QTALVELLKHKPK		1	502.3131(3)	28.51	1503.9176	1503.9137	0.0039	extract
538	544	ATEEQLK		0	409.7162(2)	11.18	817.4178	817.4181	-0.0004	43.94
545	556	TVMENFVAFVDK	M547(O)	0	472.5669(3)	35.83	1414.6788	1414.6803	-0.0015	45.06
				0	700.3511(2)	41.03	1398.6876	1398.6853	0.0022	67.63
			M547(O)	0	708.3503(2)	55.14	1414.686	1414.6803	0.0057	52.24
545	563	TVMENFVAFVDKCCAADDK	M547(O)	1	1061.4703(2)	42.1	2120.9261	2120.9217	0.0044	81.5
			M547(O)	1	707.9814(3)	42.07	2120.9223	2120.9217	0.0006	51.97
545	573	TVMENFVAFVDKCCAADDKEACFAVEGPK	M547(O) ; C566(O)	2	1070.8147(3)	42.48	3209.4223	3209.4178	0.0045	84.74
			M547(O) ; C566(O)	2	803.3618(4)	42.44	3209.4182	3209.4178	0.0004	50.24
557	573	CCAADDKEACFAVEGPK	C557(IAM) ; C558(IAM)	1	624.264(3)	27.07	1869.7701	1869.7695	0.0006	67.45
			C557(IAM) ; C558(IAM) ; C566(IAM)	1	643.2703(3)	25.34	1926.7891	1926.791	-0.0019	69.45
			C558(IAM) ; C566(IAM)	1	935.8939(2)	26.22	1869.7733	1869.7695	0.0037	91.48
			C557(IAM) ; C558(IAM) ; C566(IAM)	1	964.4043(2)	25.71	1926.794	1926.791	0.003	103.04

start	end	pep_seq	modification site	pep_miss	m/z (z)	RT (min)	exp_mass	theo_mass	delta Da (exp-theo)	pep score
564	573	EACFAVEGPK		0	525.7496(2)	27.82	1049.4846	1049.4852	-0.0006	42.89
			C566(O2)	0	541.7444(2)	27.22	1081.4743	1081.475	-0.0007	38.66
			C566(O3)	0	549.7422(2)	27.63	1097.4699	1097.4699	0	70.4
564	573	EACFAVEGPK	C566(IAM)	0	554.2605(2)	26.16	1106.5064	1106.5066	-0.0002	65.38
574	583	LVVSTQTALA		0	501.7953(2)	31.36	1001.5761	1001.5757	0.0004	51.97

*manually extracted and confirmed by CID-MS^2
